# Community Theories of Change: Linking Environmental Justice to Sustainability through Stakeholder Perceptions in Milwaukee (WI, USA)

**DOI:** 10.3390/ijerph13100979

**Published:** 2016-09-30

**Authors:** Kaitlyn Hornik, Bethany Cutts, Andrew Greenlee

**Affiliations:** 1 Department of Natural Resources and Environmental Sciences, University of Illinois Urbana-Champaign, 1102 S. Goodwin Ave., Urbana, IL 61801, USA; 2 Department of Parks, Recreation and Tourism Management, North Carolina State University, 2820 Faucette Dr., Raleigh, NC 27695, USA; bbcutts@ncsu.edu; 3 Department of Urban and Regional Planning, University of Illinois Urbana-Champaign, 611 Lorado Taft Dr., Champaign, IL 61820, USA; agreen4@illinois.edu

**Keywords:** production of injustice, socioecological interactions, perceptions, interviews

## Abstract

Environmental justice and sustainability are compatible lenses, yet action toward equity is often missing from urban sustainability initiatives. This study aims to assess the cohesion of these frameworks in practice. To do this, we parse individuals’ theories of change, or how they identify and propose to resolve environmental injustices in the pursuit of sustainability. We posit that these theories of change are comprised of three main components: (1) perceived environmental benefits and burdens; (2) the causal pathways of environmental and social injustice; and (3) visions for positive change. Drawing from 35 stakeholder interviews in Milwaukee (WI, USA) we examine individual and institutional perspectives on environmental and social change and their links to the production of injustice. Our findings reveal that participants do not distinguish between environmental and social injustices. Instead, both social and environmental factors are implicated in injustice. Furthermore, we identify two mental maps for how social and economic change reproduce injustice. These findings suggest the need to reorient how urban injustice is considered and make efforts to acknowledge how a diversity of operational theories of change could either be divisive or could bring environmental justice and sustainability initiatives together.

## 1. Introduction

As environmental justice has become an institutional imperative, there is a greater need to examine how diverse stakeholder groups construct *theories of change*. For the purposes of this paper, we define theory of change as the underlying mental model for expectations of change toward justice and sustainability. To operationalize these theories, stakeholders define concern for environmental injustices, the processes that they see as contributing to injustice, and resources that can be brought to bear in order to achieve the changes they articulate. Our goal is to draw out relevant elements of different theories and to highlight similarities and differences between them [1].

### 1.1. Background

The environmental justice movement and research agenda has a long history of uncovering distributional and procedural injustices in urban areas [2,3,4]. Environmental justice activism has focused on resisting the siting of hazardous facilities in low-income communities of color and on efforts to remediate, relocate, or otherwise compensate communities affected by pollution [5,6,7,8]. Consequently, there is an abundant body of literature that has examined the effectiveness and consequences of social movement action [6,7,8,9,10]. Over time, activism for environmental justice has given way to institutional imperatives to consider environmental (in)justice in environmental planning efforts. For instance, at the federal level, the U.S. Department of Housing and Urban Development in late 2015 adopted a new set of standards supporting its new initiative of Affirmatively Furthering Fair Housing. While this determination is focused on housing interventions involving federal dollars, the spatial distribution of exposure to environmental pollutants is now included as a measure of housing equity that will be implemented nationwide. In many instances, the institutionalization of environmental justice, which includes the creation of formal policies as well as the transition of environmental justice organizations from political groups to official not-for-profit status, has shifted how justice is defined and enacted [11,12]. Thus, although no enforceable environmental justice standards exist, Executive Order 12898 [13] and subsequent federal and state-level strategic actions have set an expectation that environmental decisions be made using fair processes that recognize the needs of low-income communities and racial and ethnic minorities, and that prevent new and remediate existing environmental injustices [14,15].

These policy shifts often adopt a working definition of justice that diverges from the philosophically liberal concepts of justice that inspire social movement action. Research has shown that governmental actions conceived of in relation to environmental justice are more likely to support a libertarian concept of justice that ascribes responsibility for environmental health protections to individuals [16,17]. Differences in operational definitions of justice between environmental justice activists, government agents, and other stakeholders are likely to change over time and between contexts. Measures of justice and policy-based remedies are likely to diverge from the philosophical principles from which they were derived. In addition, as environmental justice increasingly becomes codified within federal, state, and local policies, complex interactions are likely to result which merit additional attention and analysis [16,17].

Part of the challenge of operationalizing and implementing principles of sustainability in practice is the loose and widely interpreted nature of the concept. It is generally accepted that sustainability honors three core tenets (environmental protection, economic development, and social equity) while also thinking about how to protect the viability of these tenets for future generations [18]. However, the implementation of sustainability initiatives vis-à-vis design and policy shows that these three qualities are not always equally upheld, in fact, one would be hard pressed to find a project that equally engages all three. Principles of sustainability create the potential to link environmental improvements and economic growth with longstanding concerns regarding lagging social equity [19,20]. The road to implementing sustainable policy is filled with “tautological traps” [21], and social equity goals are often subordinated to more easily measured economic and environmental goals [22].

Previous scholarship highlights three inherent conflicts that exist within the pursuit of urban-scale sustainability interventions—a property conflict, a resource conflict, and a development conflict [23]. First, the property conflict is manifested in regards to who should establish and maintain control of how benefits of sustainable development are allocated within space. Should existing residents be guaranteed benefits associated with sustainable development or should market forces determine how benefits are allocated? In practice this conflict is often shown within price premiums for land and property that may currently, or in the future, be managed with sustainable development principles. Such improvements can essentially price existing stakeholders out of accessing benefits in their communities [24,25]. Second, the resource conflict is demonstrated through tensions regarding how to value the natural environment—with respect to development, does it have a value in and of itself, or should the value of natural resources be viewed as a substrate for urban development? Essentially, this conflict pits the conservation of nature against the prospective benefits of economic growth. Third, the development conflict embodies questions of whether development projects can adequately balance social equity and environmental concerns at the same time. While theories of environmental justice would suggest that the social and environmental landscapes are inextricably linked, in practice, these environments are often viewed as separate for the purposes of justifying and implementing development interventions. A recent emphasis on establishing principles for livability—how principles of sustainability are experienced within everyday life and everyday interactions with the natural, built, and social environment—points to another value conflict where the needs and expectations of past users of space are pitted against those anticipated to be demanded by future users [26]. The concept of “just sustainability” coined by Agyeman, Bullard, and Evans [27,28], emphasizes the importance of incorporating social improvements to sustainability projects to help bridge the gap between environmental justice and sustainability with the unified goal of creating “healthy human habitats”.

The conflicts described above help us to understand why the implementation of sustainable development and environmental justice often remain disconnected. How stakeholders percieve injustice to be created or produced is just as important as what those injustices are understood to be, though the former is a seldom-studied factor in environmental justice literature. Scholars concerned with the implementation of sustainability goals within urban contexts are increasingly looking at the roles which institutional and community culture play in cumulatively influencing social and economic systems across multiple geographic scales [29]. This approach acknowledges the importance of interactions across scales and amongst actors in everyday environments to produce social change:

Social change, once viewed as the introduction of new technologies to “innovators” or “opinion leaders” and diffused to others is now seen as stemming from the interaction of “agents”, that is individuals with agency, interaction across boundaries to solve ongoing problems at the local level.

([28], p. 247)

At the macro level, policy and regulatory institutions devise and provide resources for frameworks for change. At the meso level, organizations articulate their stake in changes and use their power to advocate for preferred alternatives and for the redistribution of benefits towards their stakeholders. At the micro level, individual community members make decisions and adapt their everyday activities in response to change. Taken together, these multilevel dynamic social systems involve both individuals and institutions interacting as positional stakeholders [30,31] across all of these levels to produce and respond to change and form a dynamic, complex, and adaptive system [32,33]. This approach suggests that in order to deal with inherent sustainability conflicts, decision-making must be integrated across scales and amongst diverse stakeholders in order to develop locally-mediated interventions and frameworks for mitigating externalities [29]. Coping with the potential trauma of change—the restructuring of physical, social, and economic benefits and burdens—across multiple levels and amongst diverse stakeholders therefore requires a more intimate understanding of how individuals and institutions perceive the nature of change and its potential to unmake or reproduce perceived forms of injustice. Many environmental improvement efforts, such as waterway remediations, are assumed to bring social and economic changes as an inherent byproduct. However, the perceived ability of such a project to better the surrounding social and economic milieu will likely differ between stakeholders.

### 1.2. Characterizing Theories of Change

Existing research is focused on perceptions of particular outcomes or processes involved in environmental injustice [34,35]. Our study takes the next step in identifying how community members believe positive change should be made to relieve injustices by examining differences in their *theories of change*.

Stakeholders define what is considered ethical and equitable in different ways, but in most cases, their ethics regards a personal view and equity refers to the distribution and access to resources and services [35]. This variation in what people believe constitutes an environmental benefit or burden may be where this divergence begins. Given differences in perception, the problem with ethical policy-making may be due to conflicts in what is perceived as ethical, just, or fair decision-making [35] and what should constitute ethical priorities and actions. These perceptions can differ from person to person and from community to community, leading to differing and conflicting concepts of what actions are most ethical and appropriate [36]. Variation in how people envision change occurring—whether it be through different individuals, agencies, policies, or other mechanisms—offers insight into potential conflicts and barriers to achieving both just and sustainable cities [37]. Conflicting views about what should change, who should initiate change, and how they should do it could illuminate this gap in sustainability that ignores injustice.

Simply identifying environmental benefits and burdens requires flexibility given that each case of equity typically has a different priority [35]. For example, one community’s focus might be public transportation whereas another’s might be legacy industrial toxins contaminating local sediments. For these reasons, recognizing the array of equity priorities at play is an important first step in understanding theories of change. Differences in scale can result in conflict over what is considered an ethical policy [34]. Beyond perceptions of inequity, it is important to consider perceptions of how injustice is created and what can be done to overcome environmental injustice. This is necessary to mediate potential conflicts that may occur in pursuit of urban justice and sustainability, as it is defined for multiple stakeholders [29].

## 2. Case Study—Milwaukee, Wisconsin

To understand how people operationalize the connection between environmental justice and sustainability, we analyze stakeholder perspectives in Milwaukee, WI, USA. We chose to engage with interview participants specifically around water resources. Water provided a useful template because it is central to both environmental injustice and sustainability in Milwaukee. Current work to clean up water pollution [38] provided an opportunity to engage with government agencies, non-profit organizations, and diverse citizen stakeholders thinking about environmental improvements in the context of larger social and ecological change.

**Figure 1:**
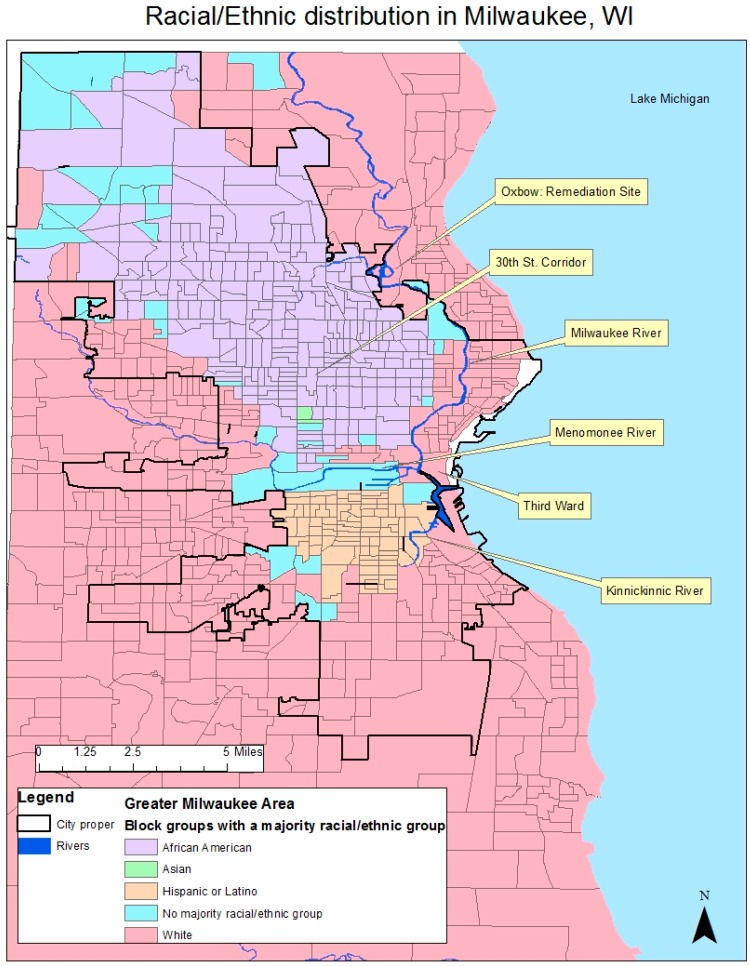
Shows the racial and ethnic makeup by census block group. To be considered a majority, over 50% of the population must be represented by one racial/ethnic group.

Located at the intersection of the Milwaukee, Kinnickinnic, and Menomonee Rivers and the shore of Lake Michigan (Figure 1), access to navigable waterways played a large role in Milwaukee’s growth as a Euro-American city, while also setting the template for patterns of racial segregation and pollution. The Milwaukee River served as a dividing line, separating sections of the city originally settled by French colonial traders who intentionally misaligned the streets to inhibit transportation of goods and people [39]. The misaligned streets, divided by the river, paved the way for discriminatory redlining resulting in heated civil rights demonstrations through the 1960s [40]. Subsequent deindustrialization in combination with “suburban supremacy” further entrenched racial disparities in terms of who bore the environmental burden of water pollution generated by the (now closed) factories [39]. Through the 1980’s the construction of divisive freeways, relatively unsuccessful urban renewal projects, white flight, suburban sprawl, and other issues common to Great Lakes Rust Belt cities further entrenched environmental injustices in Milwaukee [39,41]. Human and environmental health have been seriously threatened from industrial disinvestment, legacy pollutants, and antiquated infrastructure, all contributing to the stark socioeconomic disparities throughout the city [42,43].

With support from federal, state, and local governments, efforts to remove contaminants and restore aquatic habitat are now at the center of urban sustainability initiatives in Milwaukee. Presently, the city is undergoing robust revitalization efforts with a focus on waterways as an amenity rather than for industrial dumping or transportation. Revitalization efforts are most evident throughout the Third Ward (Figure 1) and other industrial neighborhoods undergoing commercialization with boutiques, waterfront cafes, and luxury lofted apartments [44]. Rapid revitalization efforts by the city bring opportunities to consider historical patterns of environmental injustice in the context of efforts toward a new “sustainable” economy and a view of water resources as amenities. While water-centered sustainability initiatives have been successful in some areas, Milwaukee still faces deeper politicized issues including funding for infrastructure and transportation improvements, social and economic inequality, racial segregation, high levels of concentrated poverty, and intense competition with other Great Lakes cities [39,41].

### Ethical Statement

All subjects gave their informed consent for inclusion before they participated in the study. The study was conducted in accordance with the Declaration of Helsinki, and the protocol was approved by the Institutional Review Board of the University of Illinois Urbana-Champaign (IRB #14431).

## 3. Methods

We conducted a series of semi-structured interviews with stakeholders associated with a stream remediation project in Milwaukee, WI, USA. The goal of the interview questions was to elicit the subject’s personal experiences and knowledge related to their theory of change (Table 1). Interviewing took place from February 2014 to February 2015.

Participants were selected to represent stakeholder groups relevant to ongoing stream remediation work in Milwaukee. Five different stakeholder types were identified. Resident stakeholders are people living in close proximity to the remediation site but with no professional interest in the project. Government officials are stakeholders affiliated with government entities with an interest or role in the remediation. Environmental and community NGOs (non-governmental organizations) are stakeholders affiliated with non-profit organizations with social or environmental-oriented missions. Community leaders are stakeholders identified by other participants or self-identified to be an influential and important voice in the community.

To reach a wider set of perspectives on the environment and social justice, we complimented initial purposive sampling with a referral sample. Initial interview contacts were asked to identify others they felt should be included in this study, and who were likely to share an opinion very different from their own. No more than two representatives from any one organization were invited to participate in the interview process to prevent overrepresentation from any one group. Using a combination of initial recruiting efforts and referrals, we reached 35 participants (Table 2). The intention of the sample was to draw across a large range of perceptions rather than to characterize any one stakeholder group completely. Therefore, no respondent was expected to represent a larger social group, but rather was intended to help distribute interviews across professional and personal characteristics likely to influence perceptions.

Interview analysis drew on a constructivist grounded theory approach and utilized open coding strategies, comprehensive memos, theme definition, and pile sorting as the primary methods of analysis [45]. Atlas.ti (Atlas.ti Scientific Software Development GmbH, Berlin, Germany) [46] was used to implement open coding strategies to build a list of emergent themes common throughout the interviews. Memos summarized the essence of the participant and their theory of change. From the memos, we identified common themes shared across two or more interview participants [45]. Each author read the memos and assigned the interview to a group based on their interpretation of the explicit and latent definition contained in the interview and summarized in the memo. Researchers then discussed what made groups of interviews similar, which then informed the title or brief description of each pile. To assure the reliability and validity of theme definition, the authors adapted pile sorting methods, attempting to re-sort the interviews into the previously defined thematic categories again and again [45]. Over several iterations of sorting, the definitions of the theme became more refined and the placement of interviews into piles consistent. This process was completed for all three topics of interest before we began to interpret the results [47].

## 4. Results and Discussion

This paper aims to highlight connections between environmental justice and sustainability. If urban sustainability efforts seek to successfully embrace the triple bottom line of economic development, environmental protection, and social equity, we need to make strides in closing the gap between effective research and implementation where there is a particular lag in social equity considerations. We posit that stakeholder’s theories of change have three components: (1) perceptions of environmental benefits and burdens; (2) production of social and environmental inequity; and (3) future visions for positive change. These elements help to identify places of unity between sustainability and environmental justice efforts. Our analysis illustrates how stakeholders establish and enact different theories of change in response to perceived environmental injustice and sustainability challenges in Milwaukee.

### 4.1. Identified Environmental Benefits and Burdens

Milwaukee stakeholders rarely differentiated between social and environmental factors implicated in the distribution of environmental benefits and burdens, but rather conceived of these as being one and the same. When thinking about injustice, respondents blurred the lines between what is considered “human” and what is considered “natural”. The idea of a human-nature binary is depicted in numerous papers and positioned as problematic for how people understand socio-ecological interactions [3,48,49,50]. However, our findings suggest that the lived experience of the production of justice and injustice, as reflected by our stakeholders, involves a series of inextricably linked social and environmental factors. Rather than picturing nature as “out there”, and not within the city, our stakeholders implicate both human and natural forces for the uneven distribution of benefits and burdens. What can this entangled web of social and environmental factors help us to understand about the potential for local environmental and social change to impact each other?

To answer this question, we first asked participants to identify environmental benefits and burdens. Environmental benefits, or factors affecting positive interactions and outcomes, included bike paths, maintained parks and natural spaces, access to water recreation opportunities, and environmental education opportunities. Environmental burdens, or factors detracting from the environment, included flooding, poorly maintained natural spaces, crime, unmaintained foreclosed homes, water pollution, physical danger related to stream channelization, and contaminated and unsafe fish for consumption. Looking at these lists, we observed very little delineation between environmental and social factors—evidence that our stakeholders perceive these factors as interacting and influencing each other. Rather than delineating the impact of environmental and social factors, stakeholders emphasized how benefits and burdens are unevenly distributed throughout the city spatially, temporally, sociodemographically, and socioeconomically. These stakeholders viewed environmental burdens as a mediating factor which played a role in allocating benefits and burdens and also influenced the degree to which they impacted community members. For instance, one participant who worked for a community NGO focused on the impact of urban blight. The interviewee (161A) believes that the lack of funding and action for neighborhood improvements has led to a severely blighted neighborhood, subsequently contributing to a despondent and dispirited state of mind. Thus, in the eyes of the interview participant, both social and environmental factors implicate the end result of a depressed mentality. From this perspective, environmental burdens are mutually constitutive of structural problems within the neighborhood alongside economic and social problems. This reflects a conception of nature which is not asocial [51] but rather conceives of nature as integral to the social fabric.

The list of benefits and burdens generated by stakeholders emphasized everyday sources of injustice over the more acute, high-impact sources typically highlighted in environmental justice literature where low-income minority communities are disproportionately burdened by polluters. The focus on everyday injustices recognizes different environmental burdens impairing a person’s ability to carry out tasks necessary to live out a “full life” [52]. For example, a community-based NGO (150A) talked about how lack of access to a grocery store within a low-income minority neighborhood put community residents in a position where they had to spend more resources to meet basic household needs rather than being able to invest time and energy in civic participation. Satisfying more pressing needs that are also more difficult to access by some parts of the community deters the ability of those people to participate in civic processes and engage in local decision-making.

Another spoke of injustices that interfere with the ability of citizens without personal means of transportation to enjoy parts of the city outside of their neighborhood, in particular, natural resources like Lake Michigan.

“Yes. Lack of mobility is a big problem. So it’s not likely that a lot of families go out of their particular neighborhood to go to a park or the river or even Lake Michigan. However, Milwaukee has a substantial amount of ball courts and small lots and parks that have gone, I guess with disrepair. So the opportunity is potentially there to invest back in the community, some of these resources. But the families that we talk to, they don’t—They rarely ever get down to Lake Michigan. We’ve talked to families that have kids that are teenagers that have never seen Lake Michigan (that live in) the city of Milwaukee. Yeah, so—do they get to experience a lot of these natural resources? No. But I think that it’s other conditions that are keeping them from enjoying the resources.”

(Community NGO, 157A)

Stakeholder characterization of environmental benefits and burdens demonstrates a shared recognition of everyday injustices in social, environmental, and economic change processes. Stakeholders share the view that these seemingly minor injustices interact in complex ways with local policy interventions. The constant neglect—Whether intentional or unintentional—To mediate these everyday injustices impedes quality of life, the ability for urban citizens to live out their own definitions of a productive and full life, and culminates in larger, more widespread inequalities. The prevalence of everyday injustices as a focus of participants is significant. Throughout the history of the environmental justice movement, the focus has typically been severe and large-scale cases of injustice. This study evidences that citizens do not identify isolated instances of injustice, but rather, they identify continued conditions that create injustice and the influence they have on people’s day-to-day lives [43,52,53,54].

Our respondents’ conception of injustice underscores a need to include everyday or chronic injustice in both the academic and applied field of environmental justice. Centering everyday injustice within environmental justice claims also highlights the potential for better integration of environmental justice concerns within sustainability initiatives. Sustainability initiatives call for a more holistic view of local economic, social, and environmental conditions [55,56], and interventions associated with this viewpoint may be better able to engage with the inherent unevenness of social and environmental benefits and burdens. For instance, improving transportation and access from lower income communities to become connected to natural spaces, jobs, and healthy grocery stores is a goal in a variety of city-wide sustainability plans [57,58,59], yet this lack of local access is also considered a form of injustice, as evidenced by participants in our study. Recasting sustainability analysis and planning through the lens of environmental justice provides an opportunity to increase the reach and effectiveness of local interventions.

### 4.2. Production of Social and Environmental Inequity

When examining our respondents’ mental maps for how change occurs over time, we saw evidence for two distinct frameworks for how institutional interventions can shape that change. Participants described change as: (a) following a linear pathway characterized by cause and effect relationships or (b) following a non-linear pathway characterized by complex interactions across multiple systems. These two mental maps are delineated by where stakeholders attribute the primary drivers of inequity. The divergence we observe in terms of how respondents view the social production of inequity has significant implications for how communities pursue procedural justice. Mental models based upon a linear sequence would privilege an incremental or ad hoc approach whereby a specific injustice is identified, a policy or action to address the injustice is developed, and a discrete list of stakeholders implement the necessary policy or action to address the injustice. Over time, a sequence of such responses will cumulatively result in positive social and environmental change, as well as a more equitable landscape. In contrast, a mental model based upon complex interactions focuses on the intricate relationships between multiple institutional stakeholders, whereby interventions elicit a complicated array of interactions and institutional responses. Delineating these two mental maps and identifying which stakeholders are predisposed to each perspective provides important insight into the potential for collective action to address injustice, as well as a lens for closer understanding about why well-intentioned interventions have in the past at times either exacerbated existing problems or simply displaced them to different locations.

In order to better understand which perspectives were associated with which stakeholders, we parse out stakeholders by their mental map of change. Table 3 displays which method each stakeholder type used to describe the production of injustice. Overall, the distribution of responses was relatively even between linear and non-linear pathways across stakeholders. However, within stakeholder types, stakeholders from community NGOs favored non-linear pathways while non-institutional community leaders favored linear pathways. While our sample does not allow us to discern whether this variance is indicative of larger differences between groups, their differentiation has important implications for affecting interventions on the urban environment. Future studies might test whether observed differences are related to differences in either (a) depth of interest and knowledge concerning the issue or (b) a general tendency to organize the world into either linear or non-linear systems.

#### 4.2.1. Production as a Linear Pathway

Eighteen participants described the production of injustice as a linear, causal, or sequential process in which one thing follows the next, in a logical order. This pathway is described and presented in a way that mimics an equation, where variables do not necessarily interact together, but rather, one thing is the product of the variables building upon each other. Interview participants responded to questions regarding environmental quality, social vulnerability, and social and environmental change by describing problems of injustice in a way that builds upon itself sequentially. For instance, interview participant 71A, a stakeholder working for an environmental NGO, discussed why waterways unsuitable for fishing, drinking, and swimming is the environmental injustice of concern. The creation and perpetuation of this problem is seen as a result of a series of discrete events. Following this logic, the city’s infrastructure is the root cause, beginning with the poor choice of implementing a combined sewer system, followed by failure to properly maintain an antiquated sewer system. The inability to maintain is due de-prioritization of this problem. This is a result of the more pressing problem of household sewage backups. Both time and financial constraints force municipalities to choose which problem to address; in this case, household sewage backups are the priority due to the extreme human health impacts of raw sewage in homes. Thus, the problem of combined sewer overflows (CSOs) is pushed down on the municipality’s to-do list. Still, societal and environmental processes are responsible for the production of injustice, and in this case is perceived to take a linear form. The process, according to 71A, can be ordered as follows: (1) poor infrastructure design; (2) lack of maintenance; (3) funds first allocated toward household sewage backups resulting from antiquated infrastructure; and the next step can be inferred as (4) funds allocated to updating infrastructure to eliminate CSOs. Relieving injustice is a matter of steps to be taken, dependent on available funds.

#### 4.2.2. Production as a Non-Linear Pathway

Fifteen participants described different non-linear interactions that create injustice. These interview participants responded to questions about environmental quality, vulnerable communities, and environmental and social change with statements that indicated that they viewed both environmental and social drivers as interacting in complex and often unpredictable ways to co-produce the problem at hand. For example, interview participant 159A, a community NGO stakeholder, demonstrates this notion:

“It’s gotten worse, just because of the lack of opportunities that they have. The lack of opportunities mixed, you know, if the person has a tough time finding a job and their house floods then they’re kind of SOL (shit out of luck). They have two really large issues they need to tackle. And so one sort of scratches the other’s back, in a sense.”

In the case of this participant, the production of injustice involves complex interactions between environmental hazards (flooding) and opportunity (jobs) which spin off negative externalities that disproportionately impact marginalized groups. At an earlier point in the interview, the respondent also implicates a lack of reliable transportation and spatial mismatch between jobs and housing [60,61] as other spatially mediated drivers of inequality. Flooding, job access, and disinvestment are the root causes and main drivers of uneven access to resources and situating marginalized populations in marginalized areas. The interactions of socio-environmental processes entered into a negative feedback loop, making the situation perpetually worse and nearly impossible to recover without major intervention.

In constructing the notion of injustice as a complex system, injustice is produced by complicated interactions between socio-environmental factors [43,48]. Participants clearly identified what they viewed as root causes and main drivers of injustice. In addition, describing the production of injustice as a complex system characterized by feedback loops underscores the importance of observing the circumstances under which positive or negative feedback is being generated so this information can be accounted for in the future. The concept of feedback loops encapsulates processes in cyclical patterns in which a variety of factors interact and influence the state of a particular system. Feedback loops help to illustrate that dynamic, linked variables constitute and can change a system [62].

In parsing out these two distinct mental models, it is natural to not only want to compare them to each other but also to judge their efficacy in understanding, predicting, and affecting change. Our primary goal, however, is not to characterize one model as being stronger or more efficacious when compared to the other, but rather to understand the application of the models to change making processes, as well as the potential implications for what happens when stakeholders with differing mental models collaborate to address a common problem. Engaging with this question can help us to unify understanding and produce a more holistic or systems thinking approach to understanding the forces at play in urban socioecological systems [63]. This can highlight interventions or solutions that address the root causes and main drivers of a problem rather than symptoms of larger forces [56].

### 4.3. Achieving Positive Change

Our analysis revealed organizations and agents that interviewees identified as able to make change toward environmental justice in Milwaukee. In discussing everyday environmental injustices, each participant’s response reflected one dominant perspective. In sum, we identified six perspectives on how to enact change to redress past environmental injustice as a part of efforts toward greater environmental sustainability. The visions to achieve positive change are: (1) government initiatives; (2) grassroots and NGOs; (3) community empowerment; (4) education; (5) personal action and outreach; and (6) economic development. Top-down government decision-making and grassroots organizations and NGOs were most prevalent whereas environmental education, personal action, economic development and market-based solutions were mentioned less frequently (Table 4). Each of the six visions are explained below.

#### 4.3.1. Government Initiatives

Eleven participants identified top-down government policies (from the city council-level or larger) as the most effective way to address everyday environmental injustices. For these participants, government buy-in was an essential element of improving environmental conditions in the city overall but particularly in low-income neighborhoods. For example, one interviewee discussed park funding to illustrate the importance of sustained government funding and leadership.

“I think the parks system has always been an extremely valuable commodity for those folks that don’t have material wealth in a state or a park-like atmosphere that are privately owned. So that means the vast majority of the population needs a well-run park system in order to have a place to go with their free time. And the ability to enjoy nature, have a picnic, relax, all of the things that maybe we have come to take for granted. We dare not do that because if these parks deteriorate then the masses won’t have a place to go.”

(Milwaukee Resident, 163A)

#### 4.3.2. Grassroots and NGOs

Seven participants identified non-governmental and other grassroots organizations as responsible for making change, particularly changes related to park maintenance and management. These respondents expressed greatest trust in NGOs to steward the public interest through political and financial fluctuations. For these respondents, groups with large volunteer bases were best positioned to advocate for and oversee meaningful changes in lower-income minority neighborhoods and to ensure upkeep for parks and natural spaces in the city.

#### 4.3.3. Community Empowerment

Six participants expressed visions for change grounded in different forms of community empowerment. In their interviews, they focused on creating more inclusive form of public participation and working closely with low-income and minority populations to enhance procedural forms of environmental justice. Participant 69A describes the importance of focusing on process in order to deliver outcomes that enhance both environmental justice and sustainability:

“Instead of building bigger and bigger and bigger and more and more and more, I think we need to scale back and realize that we are more rich when we have stronger community ties and stronger neighborhood ties and have a clean environment. Without that, we’re gonna be really unhealthy, and we’re gonna end up spending more and more money on things that we don’t need to.”

(Government official, 69A)

#### 4.3.4. Education

Five interviewees discussed environmental education and outreach as the most important way to inspire change perceived a need to provide new and “correct” information to people who, in their view, experienced environmental injustices as a result of larger, societally-driven environmental problems. In particular, they preferred interventions that provided resources to help Milwaukee’s youth learn about, connect to, and care for Milwaukee’s natural resources. In this view, a focus on youth is an opportunity to create a culture of urban environmental stewardship and civic engagement to respond to historically embedded patterns of environmental injustice and as-yet unanticipated challenges facing the environmental and economic future of the city.

#### 4.3.5. Personal Action and Outreach

Two participants expressed a vision for positive change grounded in personal responsibility and action. This view emphasized the need for people to personally seek the “correct” information in order to change their habits, attitudes, and behaviors. The emphasis on personal change demonstrates the notion that people need to take the initiative in making the change they wish to see. This view assigned the individual with responsibility for seeking information, knowledge, and resources necessary to make pro-environmental changes. Though it was infrequently the dominant mechanism for achieving change, it was a common secondary vision for change in other interviews.

#### 4.3.6. Economic Development

Two participants articulated visions for positive change rooted in economic development and other market-based processes. Their statements reflect the view that market-based environmental solutions help create incentives to invest in disadvantaged areas of the city.

### 4.4. Synthesis

Whereas participants expressed a relatively unified view of environmental injustice as an everyday experience (Section 4.1) and could be divided into two relatively even and mutually exclusive groups with respect to their views on how injustices are produced (Section 4.2), their visions for how to make change in support of environmental justice were more diverse. The diversity of responses highlights differences in the scale at which people envision change occurring.

Parsing out the different visions for positive change is critical to identifying where visions might conflict and what kinds of consequences these changes may create. For example, government interventions can be effective, but park maintenance and other noncritical services are frequently cut from city governance. Increasingly neoliberal policies and roll-back of government programs have led to the defunding of many public agencies, leaving private sector and non-profits to fill in those gaps [64,65,66]. Therefore, facilitating environmental justice and sustainability programs across a city may require environmental and community groups to supplement the duties of political leaders and policy enforcement which are being rapidly defunded in Milwaukee, WI, USA.

Similarly, relegating responsibility for positive change to either future generations (environmental education) or individuals (behavior change) ignores greater structural and larger-scale processes that need to be addressed in order to make substantial change, particularly related to equity in sustainability [67,68,69]. While relatively easy and cheap to implement, youth programs like those of Milwaukee’s Urban Ecology Center [70] may have a much larger impact over the access to education, environmental safety, and the environmental quality of Milwaukee’s natural areas when combined with procedural changes that engage youth (and the community overall) in the process of setting environmental clean-up priorities [71,72,73,74].

We observe that differences in pathways toward environmental justice do not follow stakeholder identities (Table 4). This observation is worthy of additional investigation, especially given the frequency with which stakeholder groups are used to frame research and policy analysis. As a result of our work, we hypothesize that cities (like Milwaukee, WI, USA) where theory of change sorts independently from stakeholder identity, will have greater capacity to develop and implement interventions that integrate concern for environmental justice and sustainability. This capacity comes through the ability to leverage interventions at multiple scales in order to address the full complexity of the system.

The focus on affecting change at any one level in isolation may overlook inequity at other levels. The need for interventions in inequity across many levels was recognized by interviewees. For example, the following quote illustrates why it might be problematic to concentrate on environmental justice for the city without also examining neighborhoods.

“Even in quality of life and green space, as areas get built up and gentrification might set in and people get priced out of their homes and their living spaces and they, again, don’t get to live in this area that maybe has a lot more beautiful green spaces. So it’s these outside forces that are maybe creating some great change for the environment, but then those folks don’t get to enjoy it.”

(Community NGO, 150A)

This statement highlights concerns that city-level action in isolation may result in the perpetuation of inequitable actions and outcomes. This is illuminated in much of the literature discussing the use of sustainability policies to enable the re-appropriation of nature, and subsequently the displacement of marginalized groups who would perhaps benefit most from environmental improvements [75].

Another key finding is the clear divergence in how to best initiate positive change between those who hold linear versus non-linear views of environmental injustice production (Table 5). Differences in visions for positive change between those with linear versus non-linear views suggest that the respondents’ theory of change shapes how they perceive opportunities to relate justice to environmental and economic elements of sustainability. Participants identified as having a linear method of constructing the production of injustice overwhelmingly favored top-down government decisions as the best agent of change with a few choosing economic development/market-based solutions and grassroots organizations and NGOs. Contrary to linear thinkers, those identified as using a non-linear pathway of injustice tended to favor bottom-up agents of change including education and outreach, community empowerment, and grassroots organizations and NGOs.

While different stakeholder types do not show distinct separation between linear and non-linear thinking (Table 4), linear and non-linear thinkers demonstrate a very clear bifurcation in the different approaches or agents responsible for making change (Table 5). This shows that stakeholder groups are diverse in both their thinking and favored agents of change. When thinking of sustainability and environmental justice as complex problems, it is beneficial to have a variety of methods working toward improvements, potentially bringing a more holistic and dynamic approach. In knowing that stakeholders are thinking at different scales and with different visions for change shows the potential to bring equal weight to the three core tenants of sustainability when working toward common goals. These findings illustrate the potential for efforts to be made across stakeholders, with diverse methods and different ways of thinking. However, success depends on whether or not these efforts are made in parallel or in tandem; opening the door for future research on how these visions and ways of thinking interact.

## 5. Conclusions

Uncovering differences and similarities in stakeholder theories of change can begin to bridge the gap between environmental justice activism and sustainability initiatives that are concurrent but not yet cohesive. In many instances, what one person considers ethical and sustainable might be considered highly unjust and unsustainable to another [35], leading to disagreement and conflict over priorities and actions. Theories of change help to understand what priorities are upheld and what people believe should be sustained [76]—environment, society, or economy—in which social equity typically falls last [20,27,77,78]. Rather than pitting one against another [23] or leaving different ethical standpoints at odds [35,36], theories of change reveal the intricacies behind the actions and priorities of activists, policy makers, residents, and NGOs alike. With the goal of creating more just and sustainable cities, it is necessary to enact multilevel and dynamic interventions that recognize a constant moving baseline [29]. This study shows that interventions across scales and stakeholders exist but may be working in parallel rather than in tandem. Bringing cohesion and mutual understanding between stakeholder priorities and acknowledging the potential for complex interactions across scales for governance can help to mitigate the potential for development conflicts that pit social equity against environmental and economic benefits.

## Figures and Tables

**Table 1:** Interview questions.

Topic	Question
Background	How would you define the physical boundaries of your local community?
Environmental burdens and benefits	How would you rate the quality of the environment in this area? What contributes to it? What detracts from it?
	How would you describe the social groups that are most vulnerable in relation to the environment?
Production of injustice	How would you describe the characteristics of (the local community)?
	What things have you seen change in (this community)? How did these changes happen?
	What types of problems has this community faced in the past? How has it dealt with those problems?
Visions for the future	What things would you like to see change about (this community)?
	Have the problems for these people gotten better or worse as (the community) has changed? Why do you think this is? Who are the leaders in this community? Who drives change?

**Table 2:** Sample composition.

Stakeholder Group	Number of Participants
Resident stakeholder	9
Government official	6
Environmental NGO (non-governmental organizations)	4
Community NGO	11
Community leader	5

**Table 3:** Stakeholder affiliation and their production of injustice pathway.

Affiliation	Linear	Non-Linear	Unidentified
Milwaukee resident	4 (44.4%)	5 (55.5%)	0
Government official	4 (66.6%)	2 (33.3%)	0
Environmental NGO	2 (50%)	1 (25%)	1 (25%)
Community NGO	4 (36.4%)	7 (63.6%)	0
Community leader	4 (80%)	0	1 (20%)
TOTAL	18 (51.4%)	15 (42.9%)	2 (0.06%)

**Table 4:** Stakeholder affiliation and their vision for positive change.

Vision for Positive Change	Stakeholder Group					
	Milwaukee Resident	Government Official	Environmental NGO	Community NGO	Community Leader	TOTAL
Government initiatives	2	2	0	4	3	11
Grassroots & NGOs	1	1	2	2	1	7
Community empowerment	1	1	1	3	0	6
Education	3	0	0	2	0	5
Personal action & outreach	0	1	1	0	0	2
Economic development	0	1	0	0	1	2
Unidentifiable	2	0	0	0	0	2
**TOTAL**	**9**	**6**	**4**	**11**	**5**	**35**

**Table 5:** Linear and non-linear thinkers and their visions for positive change.

Vision for Positive Change	Mental Model		
	Linear	Non-Linear	Unidentifiable
Government initiatives	10	1	0
Grassroots & NGOs	2	4	1
Community empowerment	1	4	1
Education	0	5	0
Personal action and outreach	1	1	0
Economic development	2	0	0
Unidentifiable	2	0	0
TOTAL	18	15	2
